# Association between prenatal antimicrobial use and offspring attention deficit hyperactivity disorder

**DOI:** 10.1371/journal.pone.0285163

**Published:** 2023-05-03

**Authors:** Jennifer K. Straughen, Alexandra R. Sitarik, Ganesa Wegienka, Christine Cole Johnson, Tisa M. Johnson-Hooper, Andrea E. Cassidy-Bushrow

**Affiliations:** 1 Department of Public Health Sciences, Henry Ford Health, Detroit, Michigan, United States of America; 2 Department of Pediatrics, Henry Ford Hospital, Detroit, Michigan, United States of America; 3 Center for Autism and Developmental Disabilities, Henry Ford Health, Detroit, Michigan, United States of America; Fudan University, CHINA

## Abstract

**Background:**

Gut-brain cross-talk may play an important role in modulating neurodevelopment. Few studies have examined the association between antimicrobials that influence infant gut microbiota assemblage and attention deficit hyperactivity disorder (ADHD).

**Objective:**

To examine the association between maternal prenatal antimicrobial use and ADHD in offspring at 10 years of age.

**Methods:**

Data are from the Wayne County Health, Environment, Allergy and Asthma Longitudinal Study, a racially and socioeconomically diverse birth cohort in metropolitan Detroit, Michigan. Maternal antimicrobial use was extracted from the medical record. ADHD diagnoses were based on parental report at the 10-year study visit. Poisson regression models with robust error variance were used to calculate risk ratios (RR). Cumulative frequency of exposure to antibiotics, and effect modification were also evaluated.

**Results:**

Among the 555 children included in the analysis, 108 were diagnosed with ADHD. During pregnancy, 54.1% of mothers used antibiotics while 18.7% used antifungals. Overall, there was no evidence of an association between prenatal antibiotic exposure and ADHD (RR [95% CI] = 0.98 [0.75, 1.29]), but there was an increased risk of ADHD among those with mothers using 3+ courses of antibiotics (RR [95%CI] = 1.58 [1.10, 2.29]). Prenatal exposure to antifungals was associated with a 1.6 times higher risk of ADHD (RR [95% CI] = 1.60 [1.19, 2.15]). In examining effect modification by child sex for antifungal use, there was no evidence of an association among females (RR [95% CI] = 0.97 [0.42, 2.23]), but among males, prenatal antifungal use was associated with 1.82 times higher risk of ADHD (RR [95% CI] = 1.82 [1.29, 2.56]).

**Conclusions:**

Maternal prenatal antifungal use and frequent prenatal antibiotic use are associated with an increased risk of ADHD in offspring at age 10. These findings highlight the importance of the prenatal environment and the need for careful use of antimicrobials.

## Introduction

Attention deficit hyperactivity disorder (ADHD) is a neurodevelopmental disorder distinguished by difficulties paying attention, impulsivity, and hyperactivity [1]. It has risen in prevalence over the last 15–20 years and recent estimates suggest that more than 8% of children in the United States are affected [2, 3]. While the causal mechanisms remain unknown, increasingly studies are suggesting that the prenatal and early-life environments may play an important role in the development of ADHD [4, 5].

Prescription medication use during pregnancy has increased over time and about 70% of women take at least 1 prescription medication during pregnancy [6]. Antimicrobials are among the most commonly used prescriptions during pregnancy [6]. Of these, antibiotics are the most frequent antimicrobial used in pregnancy, usually to treat upper respiratory, urinary tract, and sexually transmitted infections [7]. Antifungals are used to treat vaginal yeast infections, which impacts about 20% of pregnancies [8]. Broadly, antimicrobials have the potential to cross the placenta, alter the prenatal environment, and directly influence foetal development and health outcomes [9, 10]. Mounting evidence demonstrates that gut microbiota influence neurodevelopment [11–14]. The gut microbiota community and brain develop concurrently and recent studies indicate that gut-brain cross-talk may influence the trajectory of brain development with the prenatal period recognized as a potentially critical exposure window [11, 15]. Moreover, the early colonization of the infant gut is influenced by maternal microbiota and early life exposures; this early colonization may influence later microbiota diversity and ultimately neurodevelopment [16].

Despite the known impact of antimicrobials on the infant gut microbiota, the few studies that have examined prenatal exposure to antimicrobials and ADHD have reported conflicting findings [17]. For example, results from a large registry-based Canadian study suggested that prenatal antibiotic exposure was not associated with ADHD [18]. Conversely, prenatal and early life (first 2 years) exposure to antibiotics were associated with an increased risk of ADHD in a large registry-based Finish study [19].

Studies examining the association between maternal prenatal use of antifungal medications and ADHD are also lacking, although there is some evidence that systemic first trimester exposure to oral imidazole derivatives may increase the risk of neurodevelopmental defects [20]. A better understanding of the relationship between prenatal antibiotic and antifungal medications and risk of ADHD is needed. The present analysis seeks to address this gap by examining the association between prenatal antimicrobial exposure and risk of ADHD in a large racially diverse birth cohort.

## Materials and methods

### Cohort

Data for this analysis are from the Wayne County Health, Environment, Allergy and Asthma Longitudinal Study (WHEALS) birth cohort. WHEALS recruited pregnant women who were in their second trimester or later and were seeing a Henry Ford Health System obstetrics practitioner at 1 of 5 clinics [21]. All women delivered from September 2003 through December 2007, were age 21–49 years, and lived in a predefined geographic area that was selected to encourage racial and socioeconomic diversity (city of Detroit and surrounding suburban areas). Children and their parent/guardian were invited to return for a clinic visit at child age 2 years and again at child age 10 years for assessment of child health and parent/guardian completion of surveys about child health as well as sociodemographic and household characteristics. Additional details about the data elements relevant to this study are outlined in the sections below.

### Ethics approval

All participants provided written informed consent, and at the age 10 visit, children provided written informed assent. The study was approved by the Institutional Review Board at Henry Ford Health System.

### Exposure

As described previously, antimicrobial use was abstracted from maternal prenatal and delivery medical records [22]. Antibiotic use was defined as systemic antibiotic use (oral, intravenous, and intramuscular), vaginally applied antibiotics, or topically applied antibiotics at any time during pregnancy. Antifungal use was defined similarly [23]. Trimester of antimicrobial use was defined as follows: first trimester if used between 0 and less than 14 weeks gestation; second trimester if used between 14 and less than 27 completed weeks gestation; and third trimester if used from 27 weeks gestation through delivery. Number of prenatal antimicrobials used were categorized as 0, 1, 2, or 3+.

### Outcome

At the age 10 visit, the caregiver (95% the mother) reported if the child had ever been diagnosed with ADHD as well as other neurodevelopmental outcomes. Caregiver report of a “suspect” diagnosis was also classified as positive for the primary analysis. Additionally, a subset of 325 WHEALS children who had received care from Henry Ford Health System providers had their medical records abstracted for additional health information, including ADHD diagnoses and other neurodevelopmental outcomes. Given that we have previously shown near perfect agreement between self-reported and medical record-based diagnosis for ADHD (κ = 0.84, 95% CI 0.78, 0.91) in this cohort [21], the statistical analysis classified a child as having ADHD if their caregiver reported the ADHD diagnosis or if it was reported in the medical record.

Of the 1258 maternal-child pairs in the WHEALS cohort, 890 (71%) women had information on prenatal antimicrobial use. Of those, 569 of the children had information on ADHD diagnoses. Children with other neurodevelopmental outcomes (autism spectrum disorder and/or sensory processing disorder as indicted in the medical record or based on caregiver report), but not ADHD, were removed from the analysis (N = 14), leaving a total of 555 maternal-child pairs in the final analytic sample.

### Covariate definitions

During the prenatal interview, mothers self-reported race-ethnicity, insurance coverage, household income, education, marital status, previous pregnancies, smoking during pregnancy, household environmental tobacco smoke (ETS), alcohol use, indoor pets, history of asthma and allergies, and home address, which was used to define urban or suburban residence. Prenatal and delivery records were abstracted to obtain mode of delivery, body mass index (BMI) at the first prenatal visit, gestational age at delivery, and birthweight. Sex- and gestational-age adjusted birthweight z-scores were calculated using the United States population in 1999–2000 as a reference [24]. Breast feeding was maternal reported during a study visit at 1 month of age, defined as formula fed, mixed feeding, or breast fed. Race-ethnicity of child was maternal reported at 2 years of age.

### Statistical analysis

Frequencies and percentages for categorical covariates as well as means and standard deviations for continuous covariates were used to describe group differences (children included and excluded from analyses, differences by ADHD, and differences by antimicrobial use). Additionally, standardized differences (D)—defined as the difference in means or proportions divided by standard error—were used to quantify effect size, with large effect sizes considered absolute value of D > 0.2. Because selection bias due to loss to follow-up or non-response can affect the internal validity of estimates, inverse probability weighting (IPW) was used to correct for potential bias. Analytic sample inclusion was used as the outcome in a logistic regression model with the following covariates (baseline covariates hypothesized as affecting loss to follow-up): maternal age at birth, maternal race, insurance coverage, household income, marital status, maternal education, location of residence (urban vs. suburban), maternal prenatal smoking, prenatal ETS, prenatal alcohol use, prenatal indoor pets, maternal allergies and asthma, mode of delivery, parity, child sex, gestational age at birth, and birthweight. The predicted probability of inclusion for each subject was extracted from this model; weights were calculated as the inverse of the “treatment” received. In other words, if p = probability of inclusion, then IPW = 1/p for included children, and IPW = 1/(1-p) for excluded children. Covariate balance was assessed using D, with imbalance defined as absolute value > 0.20.

To evaluate the association between prenatal antimicrobial use and ADHD, risk ratios (RR) and corresponding 95% confidence intervals (CI) were obtained from Poisson regression models using a robust error variance [25]. In all models, subjects were weighted using the IPW described previously. Models were evaluated both before and after adjusting for hypothesized potential confounders, which were the following: maternal age, household income, marital status, maternal education, mother smoked during pregnancy, prenatal ETS exposure, prenatal indoor pets, maternal BMI, child sex, race-ethnicity of child, first born child, mode of delivery, breastfeeding status at 1 month, gestational age at delivery, and birthweight z-score. Because some maternal-child pairs in the analytic dataset had partial covariate missingness (11%), which we hypothesized to be missing at random, multiple imputation was performed in addition to complete-case analysis. A total of 50 imputed datasets were calculated; quality appeared to be sufficient through the examination of trace plots and variance information. Multiply imputed datasets were created using all exposure variables, outcomes, confounders, and variables thought to affect loss to follow-up as well as the IPW itself. The SAS Institute Software procedure mi with the fully conditional specification algorithm was used to generate imputed datasets; the *mianalyze* procedure was used to pool estimates.

In addition to examining any antimicrobial exposure, risk of ADHD by number of exposures (0, 1, 2, and 3+) were also calculated, but this could only be examined for antibiotic use due to small sample sizes for antifungal use (most had only 1 antifungal exposure). Systemic versus vaginal route of exposure was also examined for antifungal use (insufficient sample size for topical antifungal use); route of administration could not be examined for antibiotic use, as most exposures were systemic. Further, tests for differences in timing of antimicrobial exposure throughout pregnancy were calculated using multiple informant models, where a score test of the equality of all the trimester-specific parameter estimates was used to evaluate differences across trimesters [26]. Effect modification by child sex, mode of delivery, and breastfeeding status at 1 month were tested using interaction terms, with significance of interaction effects specified at p-value < 0.10. As a sensitivity analysis, E-values were calculated to quantify how strong an unmeasured confounder would have to be in order to negate the observed results [27]. For the primary analyses, p<0.05 was considered significant.

## Results

Among the 555 children included in the analysis, 108 (19.5%) were diagnosed with ADHD and 447 were considered neurotypical (NT). A total of 300 (54.1%) mothers used antibiotics during pregnancy while 104 (18.7%) mothers used antifungals during pregnancy; 71 women used both antibiotics and antifungals during pregnancy. A breakdown of trimester-specific use, number of exposures, and route of administration is provided in Table 1.

**Table 1:** Detailed information regarding antimicrobial use.

	Antibiotic Use (N = 300)	Antifungal Use (N = 104)
Timing of medication exposure		
1^st^ trimester only	61 (20.3%)	24 (23.1%)
2^nd^ trimester only	35 (11.7%)	27 (26.0%)
3^rd^ trimester only	102 (34.0%)	40 (38.5%)
1^st^ and 2^nd^ trimesters only	16 (5.3%)	4 (3.9%)
1^st^ and 3^rd^ trimesters only	41 (13.7%)	2 (1.9%)
2^nd^ and 3^rd^ trimesters only	27 (9.0%)	5 (4.8%)
All 3 trimesters	18 (6.0%)	2 (1.9%)
Number of medication exposures		
1	158 (52.7%)	84 (80.8%)
2	77 (25.7%)	11 (10.6%)
3+	65 (21.7%)	9 (8.7%)
Route of medication exposure		
Systemic only	262 (87.3%)	41 (39.4%)
Vaginal only	8 (2.7%)	48 (46.2%)
Topical only	7 (2.3%)	2 (1.9%)
Systemic and vaginal only	14 (4.7%)	13 (12.5%)
Systemic and topical only	8 (2.7%)	0 (0.0%)
Vaginal and topical only	0 (0.0%)	0 (0.0%)
All 3 routes	1 (0.3%)	0 (0.0%)

The children who were included in the analysis were different from those members of the cohort study who were excluded due to lack of follow-up or missing data. A comparison of children included and excluded from analyses here are shown in S1 Table. Specifically, children included in the analysis were more likely to have mothers with Health Alliance Plan coverage (a health system owned insurance plan), higher household incomes, a bachelor’s degree or more, and were less likely to have mothers who smoked prenatally. However, after weighting subjects by their IPW to mitigate selection bias, the absolute value of all standardized differences were less than 0.20 (maximum = 0.054), suggesting that balance was achieved in these covariates between included and excluded subjects.

Maternal and child characteristics and their association with ADHD are presented in Table 2. Household income, maternal education, prenatal BMI, child sex, and gestational age at delivery all demonstrated large effect sizes in terms of differences between children who developed ADHD as compared to NT children (all absolute value of D>0.2). Specifically, relative to NT children, children with ADHD had mothers who were less likely to have at least a bachelor’s degree and were more likely to have household incomes < $40,000; on average, mothers of children with an ADHD outcome had a higher prenatal BMI. Male children and those with younger mean gestational ages at birth were more likely to develop ADHD. When associations between these same characteristics and prenatal antimicrobial use were examined (Table 3), mothers who were less likely to be married or have at least a bachelor’s degree, had lower household incomes, and on average were younger and had higher BMIs were more likely to use antibiotics during pregnancy. Children of mothers who used antibiotics during pregnancy were more likely to be African American and were more likely to be formula fed at 1 month of age. Mothers who used antifungals during pregnancy were less likely to have very high household incomes ($100,000 or more), less likely to have a bachelor’s degree or higher, and were more likely to have a male child. Additionally, children of mothers who used antifungals during pregnancy were more likely to be African American.

**Table 2:** Maternal and child characteristics and their association with ADHD (N = 555).

Covariate	Level	Neurotypical N = 447	ADHD N = 108	D^a^
		N (Column %) or N, Mean ± SD		
Maternal age at birth (years)		447, 30 ± 5	108, 30 ± 5	-0.134
Household income	<$20,000	43 (9.6%)	12 (11.1%)	0.366
	$20,000-<$40,000	91 (20.4%)	30 (27.8%)	
	$40,000-<$80,000	116 (26%)	31 (28.7%)	
	$80,000-<$100,000	66 (14.8%)	12 (11.1%)	
	≥$100,000	75 (16.8%)	8 (7.4%)	
	Refused to answer	56 (12.5%)	15 (13.9%)	
Mother married	No	142 (31.8%)	43 (39.8%)	-0.169
	Yes	305 (68.2%)	65 (60.2%)	
Maternal education	<HS diploma	15 (3.4%)	2 (1.9%)	0.323
	HS diploma	65 (14.5%)	18 (16.7%)	
	Some college	193 (43.2%)	60 (55.6%)	
	≥Bachelor’s degree	174 (38.9%)	28 (25.9%)	
Mom smoked during pregnancy	No	413 (92.4%)	97 (89.8%)	0.091
	Yes	34 (7.6%)	11 (10.2%)	
Prenatal ETS exposure	No	348 (77.9%)	78 (72.2%)	0.130
	Yes	99 (22.1%)	30 (27.8%)	
Prenatal indoor pets	No	288 (64.4%)	63 (58.3%)	0.126
	Yes	159 (35.6%)	45 (41.7%)	
Maternal BMI-first measured in pregnancy (kg/m^2^)		438, 30.2 ± 7.4	107, 32.7 ± 9.3	0.303
Child sex	Male	205 (45.9%)	82 (75.9%)	-0.648
	Female	242 (54.1%)	26 (24.1%)	
Race-ethnicity of child	White	98 (21.9%)	25 (23.1%)	0.147
	African American	276 (61.7%)	71 (65.7%)	
	Other/Mixed	73 (16.3%)	12 (11.1%)	
First born child	No	279 (62.4%)	58 (53.7%)	0.177
	Yes	168 (37.6%)	50 (46.3%)	
Mode of delivery	Vaginal	279 (62.4%)	67 (62%)	0.008
	C-Section	168 (37.6%)	41 (38%)	
Gestational age at delivery (weeks)		447, 38.9 ± 1.5	108, 38.4 ± 2.1	-0.241
Birthweight z-score		433, -0.01 ± 1.0	99, 0.02 ± 1.0	0.023
Breastfeeding status at 1 month	Formula fed	88 (20.1%)	21 (20.8%)	0.025
	Mixed feeding	288 (65.9%)	66 (65.3%)	
	Breast fed	61 (14%)	14 (13.9%)	

ADHD, attention deficit hyperactivity disorder; BMI, body mass index; ETS, environmental tobacco smoke; HS, high school; SD, standard deviation.

^a^Standardized difference (D), defined as the difference in means or proportions divided by standard error. Large effect sizes are considered to be those with absolute value of D > 0.2.

**Table 3:** Maternal and child characteristics associated with prenatal antimicrobial use (N = 555).

Covariate	Level	Prenatal Antibiotic Use			Prenatal Antifungal Use		
		No N = 255	Yes N = 300	D^a^	No N = 451	Yes N = 104	D^a^
		N (Column %) or N, Mean ± SD					
Maternal age at birth (years)		255, 31 ± 5	300, 29 ± 5	-0.340	451, 30 ± 5	104, 29 ± 5	-0.154
Household income	<$20,000	17 (6.7%)	38 (12.7%)	0.472	41 (9.1%)	14 (13.5%)	0.454
	$20,000-<$40,000	49 (19.2%)	72 (24%)		103 (22.8%)	18 (17.3%)	
	$40,000-<$80,000	68 (26.7%)	79 (26.3%)		116 (25.7%)	31 (29.8%)	
	$80,000-<$100,000	55 (21.6%)	23 (7.7%)		63 (14%)	15 (14.4%)	
	≥$100,000	40 (15.7%)	43 (14.3%)		77 (17.1%)	6 (5.8%)	
	Refused to answer	26 (10.2%)	45 (15%)		51 (11.3%)	20 (19.2%)	
Mother married	No	65 (25.5%)	120 (40%)	-0.313	146 (32.4%)	39 (37.5%)	-0.108
	Yes	190 (74.5%)	180 (60%)		305 (67.6%)	65 (62.5%)	
Maternal education	<HS diploma	6 (2.4%)	11 (3.7%)	0.327	15 (3.3%)	2 (1.9%)	0.215
	HS diploma	27 (10.6%)	56 (18.7%)		66 (14.6%)	17 (16.3%)	
	Some college	111 (43.5%)	142 (47.3%)		198 (43.9%)	55 (52.9%)	
	≥Bachelor’s degree	111 (43.5%)	91 (30.3%)		172 (38.1%)	30 (28.8%)	
Mom smoked during pregnancy	No	237 (92.9%)	273 (91%)	0.072	413 (91.6%)	97 (93.3%)	-0.064
	Yes	18 (7.1%)	27 (9%)		38 (8.4%)	7 (6.7%)	
Prenatal ETS exposure	No	204 (80%)	222 (74%)	0.143	344 (76.3%)	82 (78.8%)	-0.062
	Yes	51 (20%)	78 (26%)		107 (23.7%)	22 (21.2%)	
Prenatal indoor pets	No	160 (62.7%)	191 (63.7%)	-0.019	280 (62.1%)	71 (68.3%)	-0.130
	Yes	95 (37.3%)	109 (36.3%)		171 (37.9%)	33 (31.7%)	
Maternal BMI-first measured in pregnancy (kg/m^2^)		247, 29.7 ± 7.4	298, 31.4 ± 8.1	0.221	443, 30.5 ± 7.8	102, 31.4 ± 8.0	0.113
Child sex	Male	142 (55.7%)	145 (48.3%)	0.148	221 (49%)	66 (63.5%)	-0.295
	Female	113 (44.3%)	155 (51.7%)		230 (51%)	38 (36.5%)	
Race-ethnicity of child	White	67 (26.3%)	56 (18.7%)	0.295	106 (23.5%)	17 (16.3%)	0.214
	African American	140 (54.9%)	207 (69%)		274 (60.8%)	73 (70.2%)	
	Other/Mixed	48 (18.8%)	37 (12.3%)		71 (15.7%)	14 (13.5%)	
First born child	No	163 (63.9%)	174 (58%)	0.122	270 (59.9%)	67 (64.4%)	-0.094
	Yes	92 (36.1%)	126 (42%)		181 (40.1%)	37 (35.6%)	
Mode of delivery	Vaginal	153 (60%)	193 (64.3%)	-0.089	285 (63.2%)	61 (58.7%)	0.093
	C-Section	102 (40%)	107 (35.7%)		166 (36.8%)	43 (41.3%)	
Breastfeeding status at 1 month	Formula fed	40 (16.1%)	69 (23.9%)	0.215	86 (19.6%)	23 (23%)	0.107
	Mixed feeding	169 (67.9%)	185 (64%)		288 (65.8%)	66 (66%)	
	Breast fed	40 (16.1%)	35 (12.1%)		64 (14.6%)	11 (11%)	
Gestational age at delivery (weeks)		255, 38.9 ± 1.6	300, 38.7 ± 1.7	-0.087	451, 38.7 ± 1.8	104, 38.9 ± 1.3	0.094
Birthweight z-score		244, 0.06 ± 1.0	288, -0.05 ± 1.0	-0.111	436, -0.02 ± 1.0	96, 0.08 ± 1.0	0.091

BMI, body mass index; ETS, environmental tobacco smoke; HS, high school; SD, standard deviation.

^a^Standardized difference (D), defined as the difference in means or proportions divided by standard error. Large effect sizes are considered to be those with absolute value of D > 0.2.

The association between maternal prenatal antimicrobial use and ADHD is shown in Table 4. Among children exposed to prenatal antibiotics, 60 (20%) developed ADHD, compared to 48 (18.8%) unexposed children. Prior to covariate adjustment but accounting for selection bias, no evidence of an association was found for prenatal antibiotic exposure (Model 1; RR [95% CI] = 0.95 [0.65, 1.38]). Results were similar after adjusting for potential confounders in both complete-case (Model 2; RR [95% CI] = 1.08 [0.73, 1.61]) and multiple imputation (Model 3; RR [95% CI] = 0.98 [0.75, 1.29]) analyses. However, risk of ADHD was higher among those with prenatal antifungal exposure (32 [30.8%]), than their unexposed counterparts (76 [16.9%]). The final model indicated that compared to children of mothers who did not use antifungals during pregnancy, children of mothers who did had 1.6 times higher risk of ADHD (Model 3; RR [95% CI] = 1.60 [1.19, 2.15]). The potential impact of unmeasured confounding was assessed using E-values. The observed RR of 1.6 could be explained away by an unmeasured confounder that was associated with both prenatal antifungal use and ADHD by a RR of 2.58-fold each, above and beyond the measured confounders, but weaker confounding could not do so; the CI could be moved to include the null by an unmeasured confounder that was associated with both prenatal antifungal use and ADHD by a RR of 1.67-fold each (beyond the measured confounders).

**Table 4:** Adjusted and unadjusted models examining the association between prenatal antimicrobial use and ADHD.

Exposure		N (%) of Children with ADHD	Model 1^a^	Model 2^b^	Model 3^c^
			RR^d^ (95% CI)		
Prenatal antibiotic use	No	48 (18.8%)	1.00 (Reference)	1.00 (Reference)	1.00 (Reference)
	Yes	60 (20.0%)	0.95 (0.65, 1.38)	1.08 (0.73, 1.61)	0.98 (0.75, 1.29)
Prenatal antifungal use	No	76 (16.9%)	1.00 (Reference)	1.00 (Reference)	1.00 (Reference)
	Yes	32 (30.8%)	1.81 (1.24, 2.65)	1.81 (1.20, 2.71)	1.60 (1.19, 2.15)

ADHD, attention deficit hyperactivity disorder.

^a^Inverse probability weights; unadjusted; complete-case estimate.

^b^Inverse probability weights; adjusted for maternal age, household income, marital status, maternal education, mom smoked during pregnancy, prenatal environmental tobacco smoke exposure, prenatal indoor pets, maternal body mass index, child sex, race-ethnicity of child, first born child, mode of delivery, breastfeeding status at 1 month, gestational age at delivery, and birthweight z-score; complete-case estimate.

^c^Inverse probability weights; adjusted for maternal age, household income, marital status, maternal education, mom smoked during pregnancy, prenatal environmental tobacco smoke exposure, prenatal indoor pets, maternal body mass index, child sex, race-ethnicity of child, first born child, mode of delivery, breastfeeding status at 1 month, gestational age at delivery, and birthweight z-score; multiple imputation estimate.

^d^Risk ratio (RR) is interpreted as ratio of the probability of attention deficit hyperactivity disorder in offspring, comparing women who did and did not use the specified antimicrobial prenatally.

Additionally, we hypothesized that child sex, mode of delivery, and breastfeeding status at 1 month may modify these effects (Table 5). Interaction p-values failed to reach statistical significance in all cases, except for a modifying effect of child sex in the association between prenatal antifungal use and ADHD (interaction p = 0.076). Specifically, no evidence of an association was found among females (RR [95% CI] = 0.97 [0.42, 2.23]), but among males, prenatal antifungal use was associated with 1.82 times higher risk of ADHD (RR [95% CI] = 1.82 [1.29, 2.56]). The RR among males has E-values of 3.04 and 1.90 for the point estimate and the CI, respectively.

**Table 5:** Evaluating effect modification in the association between prenatal antimicrobial use and ADHD.

Exposure	Effect Modifier	Interaction p-value^a^
Prenatal antibiotic use	Child sex	0.207
	Mode of delivery	0.945
	Any breast feeding at 1 month	0.473
Prenatal antifungal use	Child sex	0.076^b^
	Mode of delivery	0.106
	Any breast feeding at 1 month	0.849

ADHD, attention deficit hyperactivity disorder.

^a^After inverse probability weighting; adjusted for maternal age, household income, marital status, maternal education, mom smoked during pregnancy, prenatal environmental tobacco smoke exposure, prenatal indoor pets, maternal body mass index, child sex, race-ethnicity of child, first born child, mode of delivery, breastfeeding status at 1 month, gestational age at delivery, and birthweight z-score; multiple imputation analysis.

^b^Among females: risk ratio (95% CI) = 0.97 (0.42, 2.23); among males: risk ratio (95% CI) = 1.82 (1.29, 2.56).

When timing of exposure was examined (Table 6), no evidence for a trimester-specific effect was found for antibiotic or antifungal exposure (all interaction p-values ≥ 0.50). Of note, while the 95% CI for second trimester antifungal use was > 1.0, the point estimate and 95% CI for the effect of antifungals overlapped over each trimester. Additionally, a dose-response relationship between number of antibiotic exposures and ADHD was observed, where 3 or more prenatal antibiotic exposures was associated with an increased risk of ADHD (Model 3; RR [95% CI] = 1.58 [1.10, 2.29]), but a lower number of exposures was not. Most women only had 1 antifungal exposure (80.8%, Table 1); therefore, cumulative frequency of antifungal exposures could not be examined. When route of antifungal exposure was examined, vaginal exposure, but not systemic exposure, was associated with an increased risk of ADHD (Model 3; RR [95% CI] = 1.47 [1.04, 2.09]).

**Table 6:** Association between trimester, route, and number of exposures to prenatal antimicrobials and ADHD.

Exposure		N (%) of Children with ADHD	Model 1^a^	Model 2^b^	Model 3^c^
			RR^d^ (95% CI)		
**Prenatal antibiotic use by trimester**					
1^st^ trimester	No	79 (18.9%)	1.00 (Reference)	1.00 (Reference)	---^e^
	Yes	29 (21.3%)	1.12 (0.74, 1.69)	0.98 (0.64, 1.50)	
2^nd^ trimester	No	84 (18.3%)	1.00 (Reference)	1.00 (Reference)	
	Yes	24 (25.0%)	1.26 (0.82, 1.93)	1.38 (0.92, 2.06)	
3^rd^ trimester	No	68 (18.5%)	1.00 (Reference)	1.00 (Reference)	
	Yes	40 (21.3%)	1.06 (0.72, 1.56)	1.17 (0.80, 1.72)	
Interaction p-value^f^			0.82	0.50	
**Prenatal antifungal use by trimester**					
1^st^ trimester	No	99 (18.9%)	1.00 (Reference)	1.00 (Reference)	---^e^
	Yes	9 (28.1%)	1.38 (0.74, 2.58)	1.61 (0.87, 2.96)	
2^nd^ trimester	No	95 (18.4%)	1.00 (Reference)	1.00 (Reference)	
	Yes	13 (34.2%)	1.76 (1.06, 2.92)	1.66 (1.01, 2.72)	
3^rd^ trimester	No	94 (18.6%)	1.00 (Reference)	1.00 (Reference)	
	Yes	14 (28.6%)	1.66 (1.01, 2.75)	1.54 (0.96, 2.47)	
Interaction p-value^f^			0.81	0.97	
**Number of prenatal antibiotic exposures**					
	0	48 (18.8%)	1.00 (Reference)	1.00 (Reference)	1.00 (Reference)
	1	22 (13.9%)	0.66 (0.40, 1.09)	0.82 (0.47, 1.42)	0.73 (0.51, 1.03)
	2	15 (19.5%)	0.90 (0.51, 1.58)	1.16 (0.67, 2.01)	1.02 (0.67, 1.55)
	3+	23 (35.4%)	1.63 (1.01, 2.63)	1.66 (0.96, 2.85)	1.58 (1.10, 2.29)
**Route of antifungal exposure**					
Systemic	No	94 (18.8%)	1.00 (Reference)	1.00 (Reference)	1.00 (Reference)
	Yes	14 (25.9%)	1.43 (0.85, 2.39)	1.41 (0.86, 2.32)	1.23 (0.81, 1.85)
Vaginal	No	89 (18.0%)	1.00 (Reference)	1.00 (Reference)	1.00 (Reference)
	Yes	19 (31.2%)	1.61 (1.02, 2.54)	1.67 (1.03, 2.70)	1.47 (1.04, 2.09)

ADHD, attention deficit hyperactivity disorder.

^a^Inverse probability weights; unadjusted; complete-case estimate.

^b^Inverse probability weights; adjusted for maternal age, household income, marital status, maternal education, mom smoked during pregnancy, prenatal environmental tobacco smoke exposure, prenatal indoor pets, maternal body mass index, child sex, race-ethnicity of child, first born child, mode of delivery, breastfeeding status at 1 month, gestational age at delivery, and birthweight z-score; complete-case estimate.

^c^Inverse probability weights; adjusted for maternal age, household income, marital status, maternal education, mom smoked during pregnancy, prenatal environmental tobacco smoke exposure, prenatal indoor pets, maternal body mass index, child sex, race-ethnicity of child, first born child, mode of delivery, breastfeeding status at 1 month, gestational age at delivery, and birthweight z-score; multiple imputation estimate.

^d^Risk ratio (RR) is interpreted as ratio of the probability of attention deficit hyperactivity disorder in offspring, in comparator group vs. reference group.

^e^Multiple imputation estimates unable to be calculated for multiple informant models.

^f^Test for evidence of a trimester-specific effect calculated by multiple informant models.

## Discussion

We found that maternal prenatal antifungal use was associated with increased risk of ADHD in offspring. Results also suggested that the effect was restricted to males, where prenatal antifungal use was associated with 1.82 times higher risk of ADHD. Additionally, the effect appeared to be primarily driven by exposure to vaginal antifungals. Though an overall association was not detected for prenatal antibiotic use, an increased risk of offspring ADHD was observed among women who had 3 or more exposures to antibiotic medications during pregnancy. This study presents evidence supporting the importance of the prenatal environment in determining postnatal child health outcomes such as ADHD. Furthermore, the fact that the findings were restricted to males is aligned with observations of a higher prevalence of ADHD among males [3].

Strengths of this study include its prospective design and a diverse unselected study population with detailed prenatal exposures. In addition, data on antimicrobial use and last menstrual period was extracted from the medical record. As such, this data is not subject to recall bias.

Our analysis considered several potential confounders and sub-analyses of E-values suggest that our main findings are robust.

While we know the antimicrobials were prescribed, it is unknown if the medications were filled or taken as prescribed. In addition, over-the-counter antifungals are not captured, thus exposures may be underestimated. Additionally, inaccuracies in pregnancy dating could have caused inaccuracies in the trimester specific exposure analyses. Finally, as we reported elsewhere [22], data on prenatal antiviral use was only available in a subset of women and was relatively uncommon (~5.6%), thus we did not consider antiviral use an exposure in the current study. Future studies will need to examine the use of antivirals as well as maternal use of other medications or in combination with those studied here. Despite the high level of agreement between parent or caregiver reported diagnosis of ADHD and medical record-based diagnosis of ADHD, misclassification of ADHD status is possible. Finally, we did not have information on the family history of ADHD and any potential underlying genetic risk factors will need to be considered in future work.

There is very limited data about prenatal exposure to antifungals and ADHD. Although not focused on the prenatal period, one large Danish study utilizing the Danish National Patient Registry found an increased risk of ADHD in those treated with anti-infectives (including antimycotics) (hazard ratio [95% CI] = 2.09 [1.78, 2.46]) [28]. We did not have data on antifungal use in the offspring. However, additional findings can be extrapolated from a few studies that examine the fungal gut microbiota and neurodevelopment which collectively suggest that children with autism spectrum disorder have *Candida* overgrowth [29, 30]; in a cross-sectional study of children ages 3–17, the relative abundance of *Candida* was nearly 2 times higher in children with autism spectrum disorder compared to neurotypical children [31]. Prenatal exposure to antifungal use could cause gut dysbiosis by altering the maternal gut microbiota and the development of the microbiome in offspring with impacts on the gut-brain axis. In addition to altered microbiome, antifungal use may also have direct effects on neurodevelopment; however, there are limited studies examining the risks of prenatal antifungal exposure and outcomes outside the perinatal period. Systemic exposures can cross the placenta and enter the foetal bloodstream, whereas many topical applications approved for use in pregnancy are only absorbed to a limited extent [8]. In this analysis, we found a statistically significant association with vaginal application and not systemic use, but the magnitude of the effect sizes were similar, suggesting additional studies in larger samples are needed. Drug interactions are also a possible explanation; an analysis of the Hungarian Case-Control Surveillance of Congenital Abnormalities data found that treatment with a combination of antifungals (metronidazole and miconazole) was associated with poly-syndactyly, whereas individually they were not associated with an increased risk of poly-syndactyly [32]. Our analysis may have been underpowered to examine trimester-specific antifungal exposures, but trimester-stratified models did suggest that the association between antifungal use and ADHD remained in both the second and third trimesters.

Previous studies support our results which suggest that infrequent prenatal antibiotic use is not associated with ADHD. Hamad et al. examined the association between antibiotic exposure in the first year of life (defined as 1 or more prescriptions filled) and ADHD using a matched-cohort and sibling cohort design [33]. Similar to our study, Hamad et al. did not find an association between ADHD and antibiotic exposure [33]. However, they did find that a high frequency or long duration of exposure was associated with ADHD using the matched cohort design only. We also found an association between higher number of prenatal antibiotic exposures (3+) and ADHD. We did not have duration of exposure available for this analysis, rendering direct comparisons with the aforementioned study difficult. Additional studies examining the association between duration and type of prenatal antibiotic exposure are warranted.

Despite the suggestive findings, it is also possible that the maternal infection that rendered the use of the antimicrobials, and not the effects of the antimicrobial itself, contributed to the increased risk of ADHD. Such alternative hypotheses are feasible as infections can alter the type and distribution of immune and inflammatory cells, which can impact foetal and placental development [34]. For example, Gustavson et al. found that first trimester maternal fever was associated with ADHD diagnosis in offspring [35]. In this study, antifungals were primarily used to treat yeast infections, which are associated with *Candida* overgrowth, worse ADHD symptoms [36], and other psychiatric disorders [37]. While maternal *Candida* colonization is associated with offspring colonization, few offspring actually become colonized, so maternal antifungal use is unlikely to be associated with use in neonates [38]. Despite this, *Candida* infection in extremely low birthweight neonates is itself associated with neurodevelopment [39]. The cohort here was a community sample and was not enriched for pregnancy outcomes such as preterm birth or extremely low birthweight. In addition, one case study suggested that *Candida* infection worsened ADHD symptoms [36], which highlights the need for additional studies designed to disentangle the effects of maternal prenatal infections that warrant use of antimicrobials from the impact of antimicrobials use itself.

This study provides evidence for an association between maternal prenatal antifungal use and ADHD and that this association is restricted to male offspring. In addition, our analyses suggest that there is an association between frequent prenatal antibiotic use and ADHD. Additional studies are needed to confirm the association between prenatal antifungal use and ADHD and to elucidate potential pathways by which prenatal antifungal use might increase risk of ADHD. Future studies

## Supporting information

S1 Table Contains descriptive characteristics of maternal-child pairs included and excluded from the analysis. (DOCX)
