# Supplementary material for: Association between prenatal antimicrobial use and offspring attention deficit hyperactivity disorder
Source: PLoS One. 2023 May 3;18(5):e0285163. doi: 10.1371/journal.pone.0285163 (PMC10156013; doi:10.1371/journal.pone.0285163)
Supplement: S1 Table — (DOCX) [file pone.0285163.s001.docx]

**Supporting Information**

**S1 Table: Description of maternal-child pairs included and excluded from the analysis.**

| **Covariate** | **Level** | **Excluded**  **N=703** | **Included**  **N=555** | **D_Before_^a^** | **D_After_^b^** |
| --- | --- | --- | --- | --- | --- |
|  |  | **N (Column %) or N, Mean ± SD** | |  |  |
| Maternal age at birth |  | 703, 29 ± 5 | 555, 30 ± 5 | 0.189 | -0.002 |
| Race-ethnicity of mother | White | 154 (21.9%) | 136 (24.5%) | 0.113 | 0.041 |
|  | African American | 436 (62%) | 342 (61.6%) |  |  |
|  | Hispanic | 48 (6.8%) | 30 (5.4%) |  |  |
|  | Arabic | 36 (5.1%) | 23 (4.1%) |  |  |
|  | Other/Mixed | 29 (4.1%) | 24 (4.3%) |  |  |
| Insurance coverage | Health Alliance Plan | 217 (30.9%) | 285 (51.4%) | 0.639 | 0.047 |
|  | Other insurance | 227 (32.3%) | 204 (36.8%) |  |  |
|  | No insurance | 9 (1.3%) | 6 (1.1%) |  |  |
|  | Refused/do not know/missing | 250 (35.6%) | 60 (10.8%) |  |  |
| Household income | <$20,000 | 127 (18.1%) | 55 (9.9%) | 0.343 | 0.033 |
|  | $20,000-<$40,000 | 174 (24.8%) | 121 (21.8%) |  |  |
|  | $40,000-<$80,000 | 200 (28.4%) | 147 (26.5%) |  |  |
|  | $80,000-<$100,000 | 57 (8.1%) | 78 (14.1%) |  |  |
|  | ≥$100,000 | 65 (9.2%) | 83 (15%) |  |  |
|  | Refused to answer | 80 (11.4%) | 71 (12.8%) |  |  |
| Mother married | No | 300 (42.7%) | 185 (33.3%) | 0.193 | 0.033 |
|  | Yes | 403 (57.3%) | 370 (66.7%) |  |  |
| Maternal education | <HS diploma | 57 (8.1%) | 17 (3.1%) | 0.392 | 0.054 |
|  | HS diploma | 145 (20.6%) | 83 (15%) |  |  |
|  | Some college | 352 (50.1%) | 253 (45.6%) |  |  |
|  | ≥Bachelor’s degree | 149 (21.2%) | 202 (36.4%) |  |  |
| Location of residence | Suburban | 291 (41.4%) | 264 (47.6%) | -0.125 | -0.005 |
|  | Urban | 412 (58.6%) | 291 (52.4%) |  |  |
| Mom smoked during pregnancy | No | 598 (85.1%) | 510 (91.9%) | -0.215 | 0.001 |
|  | Yes | 105 (14.9%) | 45 (8.1%) |  |  |
| Prenatal ETS exposure | No | 485 (69%) | 426 (76.8%) | -0.175 | 0.003 |
|  | Yes | 218 (31%) | 129 (23.2%) |  |  |
| Prenatal alcohol use | No | 677 (96.6%) | 529 (95.5%) | 0.056 | 0.004 |
|  | Yes | 24 (3.4%) | 25 (4.5%) |  |  |
| Prenatal indoor dogs | No | 544 (77.4%) | 411 (74.1%) | 0.078 | -0.009 |
|  | Yes | 159 (22.6%) | 144 (25.9%) |  |  |
| Prenatal indoor cats | No | 594 (84.5%) | 461 (83.1%) | 0.039 | 0.033 |
|  | Yes | 109 (15.5%) | 94 (16.9%) |  |  |
| Maternal doctor diagnosed hay fever or allergic rhinitis | No | 591 (84.9%) | 463 (85%) | -0.005 | -0.009 |
|  | Yes | 105 (15.1%) | 82 (15%) |  |  |
| Maternal doctor diagnosed asthma | No | 565 (80.4%) | 440 (79.4%) | 0.023 | 0.009 |
|  | Yes | 138 (19.6%) | 114 (20.6%) |  |  |
| Mode of delivery | Vaginal | 438 (63%) | 346 (62.3%) | 0.023 | -0.010 |
|  | C-Section | 257 (37%) | 209 (37.7%) |  |  |
| First born child | No | 461 (65.6%) | 337 (60.7%) | 0.101 | 0.001 |
|  | Yes | 242 (34.4%) | 218 (39.3%) |  |  |
| Child sex | Male | 335 (47.7%) | 287 (51.7%) | -0.081 | 0.020 |
|  | Female | 367 (52.3%) | 268 (48.3%) |  |  |
| Gestational age at delivery (weeks) |  | 677, 38.7 ± 1.8 | 555, 38.8 ± 1.7 | 0.053 | -0.010 |
| Birthweight (grams) |  | 650, 3252 ± 560 | 532, 3368 ± 584 | 0.197 | -0.009 |

ETS, environmental tobacco smoke.

^a^Standardized difference (D), defined as the difference in means or proportions divided by standard error, before inverse probability weighting to account for selection bias.

^b^Standardized difference (D), defined as the difference in means or proportions divided by standard error, after inverse probability weighting to account for selection bias.
